# Thermal management analyses of induction motor through the combination of air-cooling and an integrated water-cooling system

**DOI:** 10.1038/s41598-023-36989-2

**Published:** 2023-06-22

**Authors:** Sameer Madhavan, Raunak Devdatta P B, Yashwanth Reddy Konda, Edison Gundabattini, Arkadiusz Mystkowski, Ryszard Palka, Marcin Wardach, Pawel Prajzendanc

**Affiliations:** 1 School of Mechanical Engineering, Vellore Institute of Technology (VIT), Vellore, Tamil Nadu 632 014 India; 2 SQL Database Administration, Infosys Technologies Limited, Gachi Bowli, Hyderabad, Telangana 500032 India; 3 Department of Thermal and Energy Engineering, School of Mechanical Engineering, Vellore Institute of Technology (VIT), Vellore, Tamil Nadu 632 014 India; 4 Department of Automatic Control and Robotics, Faculty of Electrical Engineering, Bialystok University of Technology, Wiejska 45D, 15-351 Bialystok, Poland; 5 Faculty of Electrical Engineering, West Pomeranian University of Technology in Szczecin, Sikorskiego 37, 70-313 Szczecin, Poland; 6 Faculty of Mechatronics and Electrical Engineering, Maritime University of Szczecin, Waly Chrobrego 1-2, 70-500 Szczecin, Poland

**Keywords:** Energy science and technology, Engineering, Physics

## Abstract

The correct strategy of heat management in electric machines is extremely important due to their operating costs and length of operation. In this paper, the thermal management element strategies of the induction motors are developed to assure better endurance and boost efficiency. Additionally, an extensive review of the literature was carried out in terms of cooling methods for electrical machines. As the main result, the thermal analysis of an air-cooled and large-capacity induction motor is given, considering well-known heat distribution problems. Moreover, this study also presents an integrated approach with two or more cooling strategies to be the need of the hour. A model of a 100-kW air-cooled induction motor and an improved thermal management model of the same motor were both numerically investigated, using a combination of air cooling and integrated water cooling systems to achieve a significant improvement in motor efficiency. The integrated system comprising air- and water-cooled systems are investigated using SolidWorks 2017 and ANSYS Fluent version 2021. Three different flow rates of water 5 LPM, 10 LPM and 15 LPM are analyzed and compared with a conventional air-cooled induction motor, which was validated with the available published resources. Performed analyses indicate that for different flow rates of 5 LPM, 10 LPM and 15 LPM respectively, we have obtained a reduction of temperature accordingly of 2.94%, 4.79% and 7.69%. Hence, the results indicated that an integrated induction motor is efficient in bringing down the temperature compared to air cooled induction motor.

## Introduction

**Figure 1:**
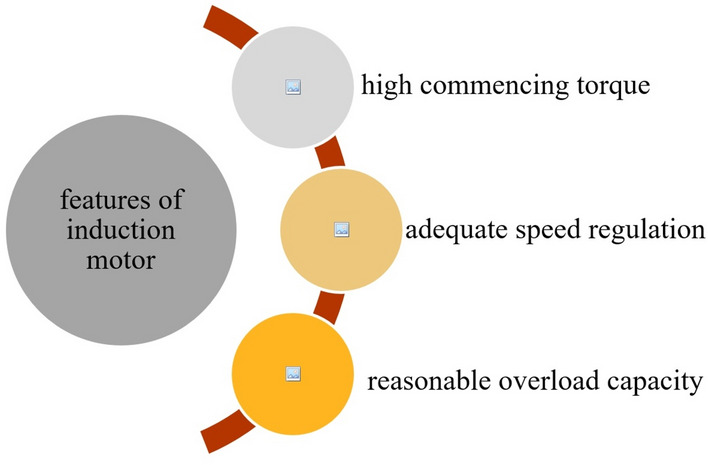
List of features of the induction motor.

The electric motor is one of the key inventions of modern engineering sciences. Electric motors are used in a variety of sectors, from home appliances to transportation, including automotive and aerospace. Induction motors (IMs) have grown in popularity in recent years due to their high starting torque, good speed control, and appropriate overload capability (Fig. 1). The induction motor not only makes your light bulb glow but also powers most of the gadgets in your home every day from a toothbrush to a Tesla car. Mechanical power is created in an IM by the contact of the magnetic fields of the stator and rotor windings. Furthermore, because rare-earth metals are in limited supply, IMs are a viable choice. However, the main disadvantage of IMs is that their lifespan and efficiency are very temperature sensitive. Induction motors consume around 40% of all electricity in the world which should make us think, that the energy management of these machines is utterly crucial.

The Arrhenius equation states that the lifespan of the entire motor is halved every time the functioning temperature is increased by 10 °C. Thus, to ensure reliability and improve machine performance, it is necessary to focus on the thermal management of IMs. Thermal analysis has garnered insufficient attention in the past, and motor developers have addressed it only peripherally based on design expertise or other sizing variables such as winding current density etc. These methods lead to applying a large factor of safety, to deal with the worst heating situations thereby leading to oversizing of the machine and in turn, increasing the cost.

### Modeling through the thermal analysis

Thermal analysis is classified into two types: analytical lumped circuits and numerical approaches. The analytical approach's key benefit is its ability to calculate quickly and precisely. However, much work must be expended to define a circuit that is exact enough to mimic the heat routes. Numerical approaches, on the other hand, are broadly classed as Computational Fluid Dynamics (CFD) and Structural Thermal Analysis (STA), both of which employ Finite Element Analysis (FEA). The numerical analysis has the benefit of enabling us to simulate the device geometry. It may, however, be difficult at times regarding system setup and computing work. The scientific papers discussed below are selected examples of thermal and electromagnetic analysis of various modern induction motors. These papers inspired the authors to undertake work on thermal phenomena in induction machines and methods of their cooling.

Pil-Wan Han^1^ investigated the thermal and electromagnetic analysis of an IM. The analytical lumped circuit method was used for thermal analysis whereas time-varying magnetic FEM was used for electromagnetic analysis. In order to properly assure overload thermal protection in any industry application, it is necessary to reliably estimate stator winding temperature. Ahmed et al.^2^ had presented a higher-order thermal network model based on in-depth heat and thermodynamic considerations. The development of the thermal model method for use in industries for thermal protection objectives benefited from the analytical solution and thermal parameter considerations.

Nair et al.^3^ predicted thermal distribution in the motor using coupled analytical and 3D numerical thermal analysis of an IM rated at 39 kW. Ying et al.^4^ analysed fully enclosed fan-cooled (TEFC) IM with the help of a 3D temperature estimation. Moon et al.^5^ studied the characteristics of the thermal flow of a TEFC IM using CFD. Todd et al.^6^ gave a transient LPTN model of an electric motor. The experimental temperature data was used with the calculated temperature obtained by the LPTN model proposed. Peter et al.^7^ studied the airflow which affects the thermal management of an electric machine using CFD.

**Figure 2:**
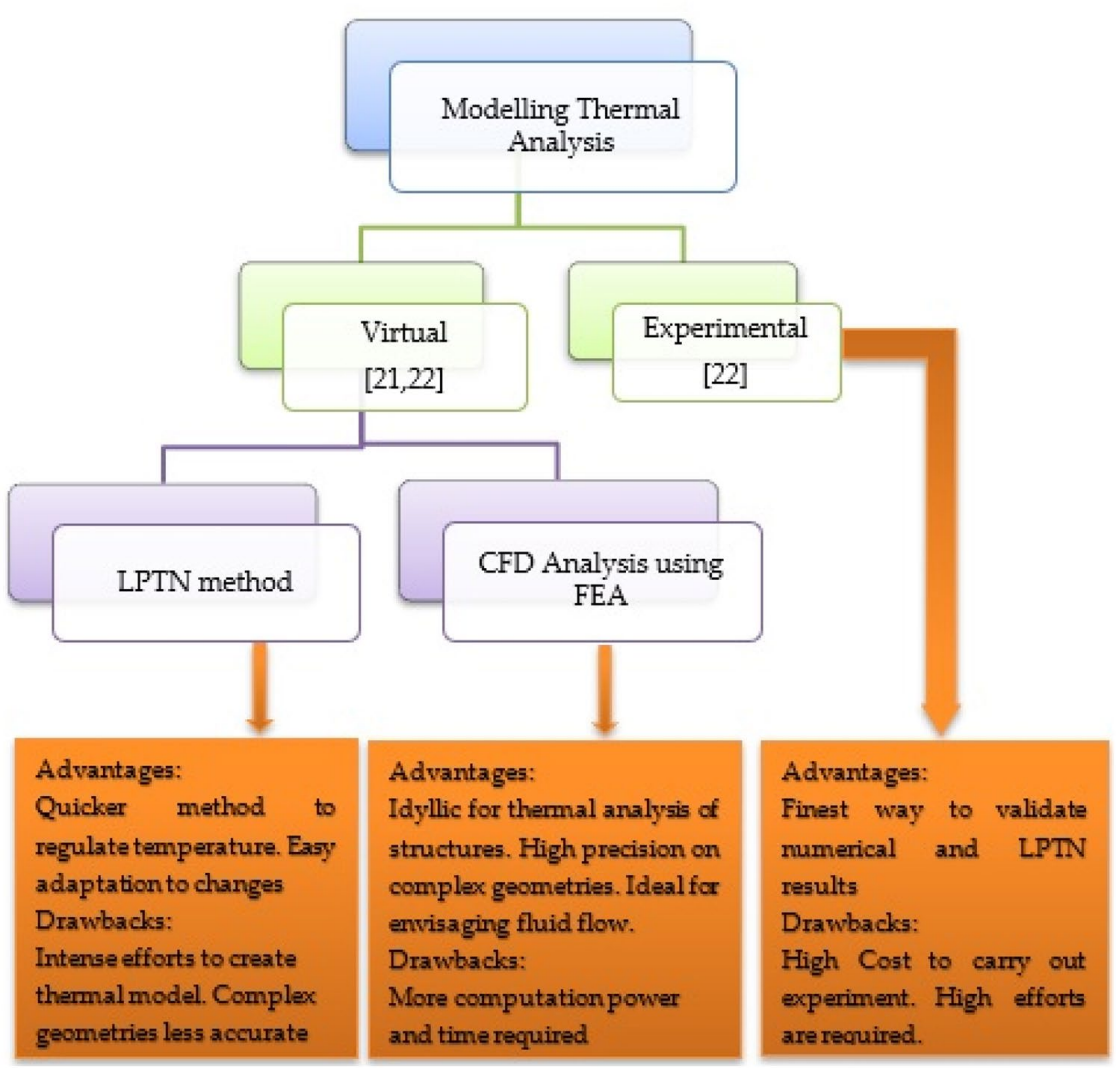
Thermal analysis of an electric motor.

Cabral et al.^8^ presented a simple thermal model of the IM, where the temperatures of the machine were obtained through the application of the heat diffusion equation in a cylinder. Nategh et al.^9^ investigated a self-ventilated traction motor system using CFD to examine the accuracy of the components for optimisation. Hence the modelling of thermal analysis in an induction motor could be envisaged using numerical and experimental investigations, see Fig. 2.

### Thermal management and the cooling schemes

Yinye et al.^10^ proposed a design for the enhancement of thermal management with the useual thermal properties of the standard materials and with the common sources of losses in the machine parts. Marco et al.^11^ proposed design criteria for the cooling systems and water jacket for the machine parts with the help of CFD and LPTN models. Yaohui et al.^12^ provided various guidelines for the selection of appropriate cooling methods and evaluating the performance in the early stages of design. Nell et al.^13^ presented coupled electromagnetic–thermal simulation using models in terms of its value range, level of detail, and computational effort for a given multiphysical problem. Zhan et al.^14^ and Kim et al.^15^ investigated the temperature distribution of an air-cooled asynchronous IM with the help of a 3D coupled field FEM. The input was calculated using a 3D eddy-current field analysis to find the joules loss and use it for thermal analysis.

#### Air-cooling

Michel et al.^16^ compared the traditional centrifugal cooling fan with different axial fan designs by carrying out simulations and experiments on them. One of the designs provided a small significant improvement in the motor efficiency while maintaining the same operational temperatures.

Lu et al.^17^ used the equivalent magnetic circuit method combined with the Boglietti’s model to estimate iron losses in induction motor spindles. The authors assumed that the distribution of magnetic flux density in any cross section inside a spindle motor is uniform. They compared their method with the results of FEM analysis and experimental model. The method can be used for rapid analysis of IM, but its accuracy is limited.

In^18^, various methods of electromagnetic field analysis in linear induction motors are presented. Among other things, a method for the power loss evaluation in the reaction rail and the temperature rise prediction method of traction linear induction motors are described. These methods can be used to improve the efficiency of energy conversion in linear induction motors.

#### Liquid-cooling

Zabdur et al.^19^ investigated cooling jackets’ performance using a 3D numerical method. The cooling jacket used water as a prime source of coolant for a 3-phase IM with great importance to the power required for pumping and the maximum temperature. Rippel et al.^20^ patented a new method of the liquid cooling system called Transverse Lamination Cooling wherein the coolant flowed transversely through a narrow region formed by apertures in every other magnetic lamination. Deriszadeh et al.^21^ experimentally investigated cooling traction motors in the automotive sector with a mixture of ethylene glycol and water. The performance at various mixtures is evaluated using CFD and 3D turbulent fluid motion analysis. Boopathi et al.^22^ investigations using simulations revealed that the water-cooled motor's temperature range (17–124 °C) was significantly smaller than the air-cooled motor's (104–250 °C). The highest temperature of the water-cooled motor with an aluminium casing was reduced by 50.4%, while the highest temperature of the water-cooled motor with a PA6GF30 casing was reduced by 48.4%. Bezyukov et al.^23^ evaluated the effect of scale formation on thermal conductivity on motor walls with liquid cooling systems. Investigations indicated that a scale layer of 1.5 mm thickness bring down the heat transmission by 30%, increases fuel consumption and reduces motor power.

#### Oil injection-cooling

Tanguy et al.^24^ experimented on an electric motor with lubrication oil as a coolant for varied flow rates, temperatures of the oil, speed of rotation and patterns of injection. A strong dependency was established between flow rates on global cooling performance. Ha et al.^25^ proposed a dripping nozzle as the spray nozzle to evenly distribute oil film to maximize the cooling performance of the motor.

#### Heat pipes and PCM-cooling

Nandy et al.^26^ analyzed the effect of *L*-shaped flat heat pipes on the performance and thermal management of an electric motor. Heat pipe evaporator parts were installed inside the motor housing or buried in the motor shaft, while condenser parts were installed cooled by circulating liquid or air. Bellettre et al.^27^ investigated a solid–liquid PCM cooling system for a motor stator working at a transient state. PCM impregnated the winding heads and due to latent heat energy storage, the hot spot temperature is lowered.

**Figure 3:**
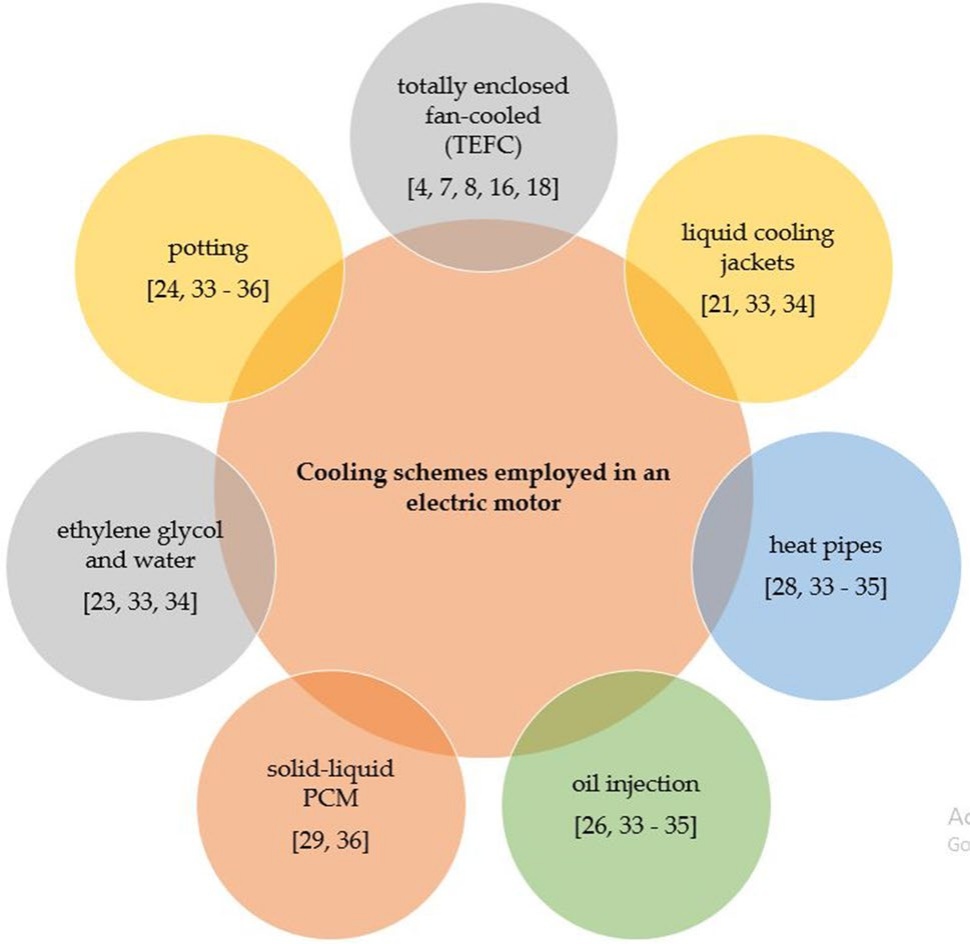
Various cooling strategies to enhance the thermal performance.

Hence, the performance and thermal management of an electric motor are estimated by employing various cooling strategies, see Fig. 3. These cooling schemes are to control the temperature on windings, laminations, winding heads, magnetic components, machine frames as well as on the end plates.

### Voids in literature and scope for improvement

Liquid cooling systems have been known for their high effectiveness in terms of heat transfer capabilities. However, an ample amount of energy is consumed to pump the coolant around the motor, thereby reducing the effective power output of the motor. On the other hand, an air-cooling system is a widely used method due to its low cost and easy-to-retrofit capabilities. But, as compared to a liquid cooling system, its effectiveness remains low. An integrated approach is necessary which can combine the high heat transfer nature of the liquid-cooled system with the low cost of the air-cooled system, without needing to consume extra power.

In this paper, thermal losses in the IM are listed and analyzed. The mechanics of the problem, as well as the heating and cooling of the induction motor, are explained in "Thermal losses in induction motors" to "Cooling strategy". The induction motor's core thermal losses are transformed into heat. Hence, this paper discusses the heat transfer mechanism inside the motor through conduction as well as forced convection. Thermal simulation of the IM through Continuity Equation, Navier–Stokes’s/Momentum Equation and Energy Equation is debriefed. The researchers conducted an analytical and numerical thermal study of an IM to assess the temperature of the stator windings only with the intention of thermal management of the electric motor. This paper mainly discusses the thermal analysis of air-cooled IMs and the thermal analysis of integrated air- and water-cooled IMs using CAD modelling and ANSYS Fluent Simulations. Also, the thermal advantage of the integrated and improved model consisting of the air- and the water-cooled system is thoroughly analyzed. As stated above, the papers listed here are not a summary of the state of the art regarding thermal phenomena and cooling of induction machines, but they signal a multitude of problems that need to be solved for the reliable operation of induction machines.

## Thermal losses in induction motors

The thermal losses are typically classified into copper losses, iron losses and friction/mechanical losses.

### Copper losses

The copper losses are the result of the Joule heating which occurs due to the resistivity of the conductors and can be quantified by^10,28^:

1 $${\dot{q}}_{g}={I}^{2}{R}_{e}=\frac{{Ve}^{2}}{{R}_{e}},$$

where *q̇*_*g*_ represents the heat generated, *I* and *V*_*e*_ are the rated current and voltage respectively and *R*_*e*_ is the resistance of the copper.

### Iron losses

The iron losses also called stray losses are the second most dominant type of loss that produces hysteresis and eddy current losses in IMs primarily caused by time-varying magnetic fields. These are quantified by the extended Steinmetz equation whose coefficients can be treated as constants or variables depending upon the operating conditions^10,28,29^.

2 $${P}_{core}=\sum {{K}_{hn}B}_{n}^{1.6}N x f +{{K}_{en}B}_{n}^{2}{\left(N x f\right)}^{2},$$

where *K*_*hn*_ is the coefficient of hysteresis loss derived from the core loss graph, *K*_*en*_ is the coefficient of eddy current loss, *N* represents the harmonic index, *B*_*n*_ and *f* represent peak flux density and frequency of the non-sinusoidal excitation, respectively. The above equation can be further simplified as follows^10,29^:

3 $${\dot{q}}_{core}={\dot{q}}_{ec}+{\dot{q}}_{h}+{\dot{q}}_{ex}={{K}_{1}B}_{n}^{2}+{{K}_{2}B}_{n}^{1.5},$$

where *K*_*1*_ and *K*_*2*_ represent the core loss coefficients and eddy current loss (*q*_*ec*_), hysteresis loss (*q*_*h*_) and excessive loss (*q*_*ex*_), respectively.

### Mechanical losses

Windage and friction losses are the two major causes of mechanical loss produced in an IM. The windage and friction losses are given as^10^,

4 $${P}_{fw}=2\times {D}^{3}\times l\times {n}^{3}\times {10}^{-6}+{K}_{fb }\times G\times n\times {10}^{-3},$$

where *n* is the rotational speed, *K*_*fb*_ is the frictional loss coefficient, *D* denotes the outer diameter of the rotor, *l* is the length of the rotor, and *G* is the weight of the rotor^10^.

## Heat transfer analysis

### Conduction

The main heat transfer mechanism inside the motor is through conduction with internal heat generation, which can be given by Poisson’s equation applicable in the present case is^30^:

5 $$\frac{{\partial }^{2}t}{\partial {x}^{2}}+\frac{{q}_{g}}{k}=0,$$

where *q*_*g*_ and *k* are the heat generation and thermal conductivity of the solid, respectively.

During operation, after a certain point of time when the motor reaches a steady state, the heat generation can be approximated as constant surface heat flux heating. Thus, the conduction inside the motor can be assumed to be a case of conduction with internal heat generation.

### Convection

The heat transfer between the fins and the ambient atmosphere is considered forced convection where the movement of the fluid is forced in a particular direction with the help of external force. Convection can be expressed as^30^:

6 $${Q}_{Conv }=hA\Delta T,$$

where *h* represents the heat transfer coefficient (W/m^2^ K), *A* is the surface area and Δ*T* is the temperature difference between the heat transfer surface and coolant normal to that surface. The Nusselt number (*Nu*), which is a measure of the ratio of convective to conductive heat transfer normal to the boundary, is chosen based on the characteristics of laminar and turbulent flows. Based upon the empirical method, the Nusselt number for turbulent flow is normally correlated with the Reynolds number and Prandtl number and is expressed as^30^:

7 $$Nu=\frac{hl}{\lambda }=f \left(Re, Pr\right),$$

where *h* is the convective heat transfer coefficient (W/m^2^ K), *l* is the characteristic length, *λ* is the thermal conductivity of the fluid (W/m K), and the Prandtl number (*Pr*) is a measure of the ratio of momentum diffusivity to heat diffusivity (or the relative thickness of the velocity and the thermal boundary layers) which is defined as^30^:

8 $$Pr=\frac{\mu {C}_{p}}{k},$$

where *k* and *c*_*p*_ are the thermal conductivity and specific heat of fluid, respectively. Generally, air and water are the most common coolant for electrical machines. The fluid properties of air and water at ambient temperature are given in Table 1.

**Table 1:** Fluid properties of water and air at 30 °C^17,22^.

Fluid properties	Water	Air
Prandtl number, ***Pr***	6.59	0.72
Density, ${\varvec{\rho}}$ (kg/m^3^)	998.00	1.20
Dynamic viscosity, ${\varvec{\mu}}$ (N s/m^2^)	9.77 × 10^–4^	1.82 × 10^–5^
Thermal conductivity, ${\varvec{\uplambda}}$ (W/m K)	0.6048	0.0256
Specific heat, C_p_ (J/kg K)	4076.00	1006.00

To analyze the forced convection across the surface of the fins, the following equation is used^30^:

9 $${Nu}_{f}={\left(\frac{1}{{\left(0.5RePr\right)}^{3}}+\frac{1}{{\left(0.664\sqrt{Re}{Pr}^{0.33}\sqrt{1+3.\frac{65}{\sqrt{Re}}}\right)}^{3}}\right)}^{-0.33}.$$

## Cooling strategy

The thermal simulation of the IM was based on the following assumptions, 3-D steady state, turbulent flow, the air is an ideal gas, negligible radiation, Newtonian fluid, incompressible fluid, no-slip condition and constant properties. Accordingly, to satisfy the conservation principles of mass, momentum, and energy in the fluid area, the following equations are used.

### Continuity equation

In general, the mass conservation equation is equal to the net flow rate of mass into the fluid element, which is determined by^31^:

10 $$\frac{\partial \rho }{\partial t }+ \nabla .\rho V=0,$$

where *V* is a velocity vector. In the case of steady flow, Eq. (10) could be modified into:

11 $$\nabla . \rho V = 0.$$

### Navier–Stokes’s/momentum equation

According to Newton’s second law, the rate of change of momentum of a fluid particle is equal to the sum of the forces acting upon it, the general momentum conservation equation can be written in vector form as^31^:

12 $$\frac{\partial (\rho V)}{\partial t}=-\nabla p+\nabla .{\tau }_{ij}+ \rho g.$$

The terms *∇p, ∇∙τ*_*ij*_ and ρ*g* in the equation above represent the pressure, viscous and gravitational force, respectively. The cooling media (air, water, oil, etc.) which are used as a coolant in machines are normally considered Newtonian in behaviour. The equation shown here is solely concerned with the linear relation between the shear stresses and velocity gradient (strain rate) normal to the direction of the shear. Considering constant viscosity and steady flow the Eq. (12) can be modified into^31^:

13 $$V\cdot \nabla \rho V=-\nabla p+\mu {\nabla }^{2}V+\rho g.$$

### Energy equation

The rate of change of energy of a fluid particle is equal to the sum of the net rate of heat contributed to the fluid particle and the net rate of work done on the fluid particle, according to the first law of thermodynamics. For a Newtonian compressible viscous flow, the energy conservation equation can be expressed as^31^:

14 $$\rho {C}_{p}\frac{\partial T}{\partial t}=\nabla \cdot \left(k\nabla T\right)+\varnothing ,$$

where *C*_*p*_ is the heat capacity at constant pressure, the term *∇* ∙ (*k∇T*) relates heat conduction through the boundaries of the fluid element, where *k* represents the thermal conductivity. The conversion of mechanical energy into heat is considered by the term $\varnothing$, (i.e., viscous dissipation function) and is given by^31^:

15 $$\varnothing =\mu \left[2{\left(\frac{\partial \mathrm{u}}{\partial \mathrm{x}}\right)}^{2}+{2\left(\frac{\partial \mathrm{v}}{\partial \mathrm{y}}\right)}^{2}+{2\left(\frac{\partial \mathrm{w}}{\partial \mathrm{y}}\right)}^{2}+{\left(\frac{\partial \mathrm{v}}{\partial \mathrm{x}}+\frac{\partial \mathrm{u}}{\partial \mathrm{y}}\right)}^{2}+{\left(\frac{\partial \mathrm{w}}{\partial \mathrm{y}}+\frac{\partial \mathrm{v}}{\partial \mathrm{z}}\right)}^{2}+{\left(\frac{\partial \mathrm{u}}{\partial \mathrm{z}}+\frac{\partial \mathrm{w}}{\partial \mathrm{x}}\right)}^{2}\right],$$

where $\rho$ is fluid density, $\mu$ is the fluid viscosity, *u*, *v* and *w*, are the respective *x*, *y*, *z* direction potentials of the fluid velocity. The term describes the conversion of mechanical energy into thermal, which can be neglected since the only importance it has is when the fluid viscosity is very high and when the velocity gradient of the fluid motions is very large. In the case of steady flow, constant specific heat and thermal conductivity, the energy equation is modified as:

16 $${C}_{p}V\cdot \nabla \rho T=2k\nabla T.$$

These governing equations are solved for laminar flow in cartesian coordinates. However, like many other engineering problems, the operation of electrical machines mainly involves turbulent flows. Consequently, these equations are modified to form the Reynolds-Averaged Navier–Stokes (RANS) approach for turbulence modelling.

## Design approach and details

### Methodology

In the present work, ANSYS Fluent 2021 software was identified to be used for the CFD simulations with appropriate boundary conditions, such as considered model: 100 kW air-cooled induction motor; rotor diameter: 80.80 mm; stator diameter: 83.56 mm (inner) and 190 mm (outer); air gap: 1.38 mm; overall length: 234 mm; number of fins: 30; thickness of fins: 3 mm.

**Figure 4:**
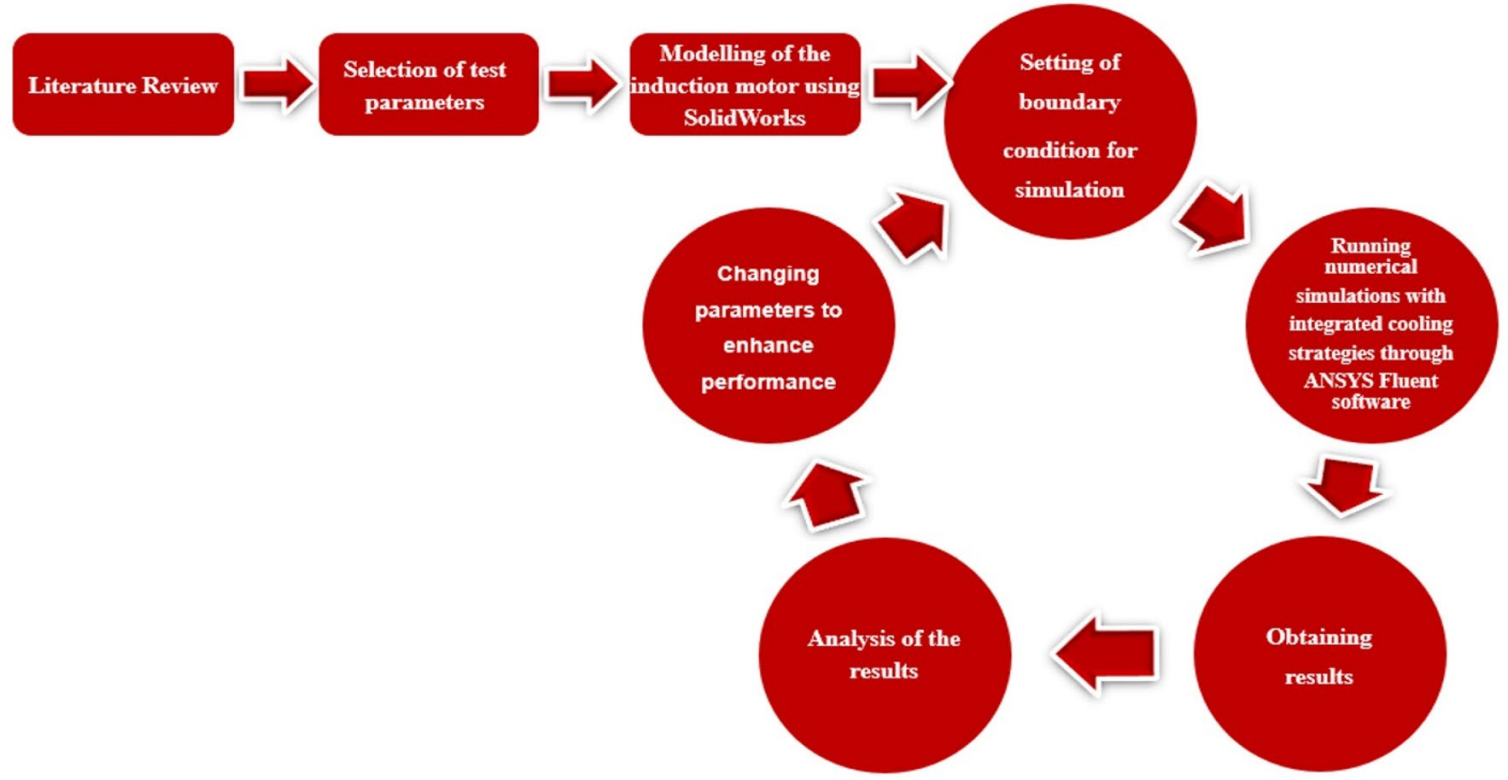
Methodology adopted in this research.

Further, the SolidWorks model of the air-cooled motor was imported into the ANSYS Fluent software and simulations were conducted. Furthermore, the validation of the obtained results was done to ensure the accuracy of the performed simulations. Also, the integrated air- and water-cooled IM is modelled using SolidWorks 2017 software and simulations using ANSYS Fluent 2021 software (Fig. 4).

### CAD modeling

The model’s design and dimensions are inspired by the Siemens aluminium series 1LA9 and modelled in the SolidWorks 2017. The model is slightly modified to cater for the needs of the simulation software. The CAD model is modified by removing unnecessary parts, fillets, chamfer, etc. are removed while simulating through ANSYS Workbench 2021.

**Figure 5:**
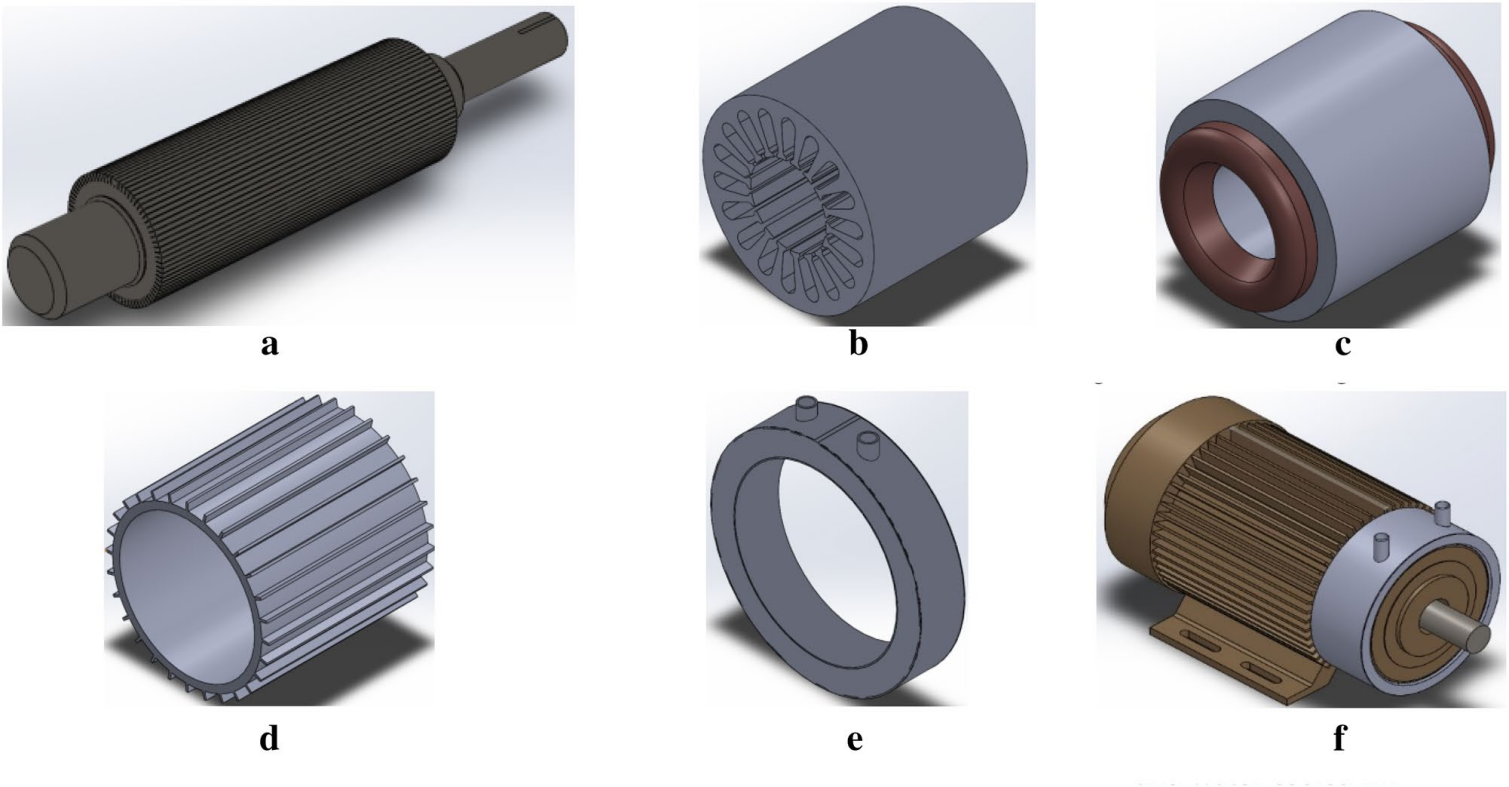
(**a**). Rotor core and shaft of the IM. (**b**) Stator core of the IM. (**c**) Stator windings of the IM. (**d**) Outer frame of the IM. (**e**) Water jacket of the IM. (**f**) Model of integrated air- and water-cooled IM.

The innovation in the design is the water jacket and the length of which is decided after the results obtained in the simulation of the first model. Several changes were made while modelling the water jacket to obtain the best possible results with the constrictions in the ANSYS. The different parts of the IM are shown in Fig. 5a–f.

### Boundary conditions

The shaft-mounted fan ensures a constant airflow velocity of 10 m/s and a temperature of 30 °C, over the fin surface. The value of velocity is randomly chosen depending on the capacity of the IM analyzed in this paper, which is larger compared to the one in literature^9^. The heat generation regions include the rotor, stator, stator winding and rotor cage bar. Material considered for the stator and rotor is steel; for windings and cage bar is copper; and for the frame and fin is aluminum. The heat generated in these areas is due to electromagnetic phenomena such as Joule’s heating where external current passes through the copper coils and due to changes in the magnetic field. The heat generation rates for different parts were gathered based on different literature available on a 100-kW IM.

The integrated air- and water-cooled IM, in addition to the above-mentioned conditions, also comprises a water jacket where different water flow rates namely 5 LPM, 10 LPM and 15 LPM were analyzed for their heat transfer abilities and pumping power requirements. Since the findings did not significantly change when the flow rate was below 5 LPM, this valve was chosen as the minimum. Furthermore, a flowrate of 15 LPM was chosen as the maximum since the pumping power increased noticeably although the temperatures continue to drop below.

### ANSYS fluent simulations

#### Geometry

The different models of the IM were imported into ANSYS Fluent and further edited using ANSYS Design Modeler. A box-shaped enclosure of dimensions 0.3 × 0.3 × 0.5 m is further constructed around the IM to analyze the flow of air movement around the motor and to study the heat dissipation into the atmosphere. A similar analysis is done for the integrated air-cooled and water-cooled IM.

#### Meshing

**Figure 6:**
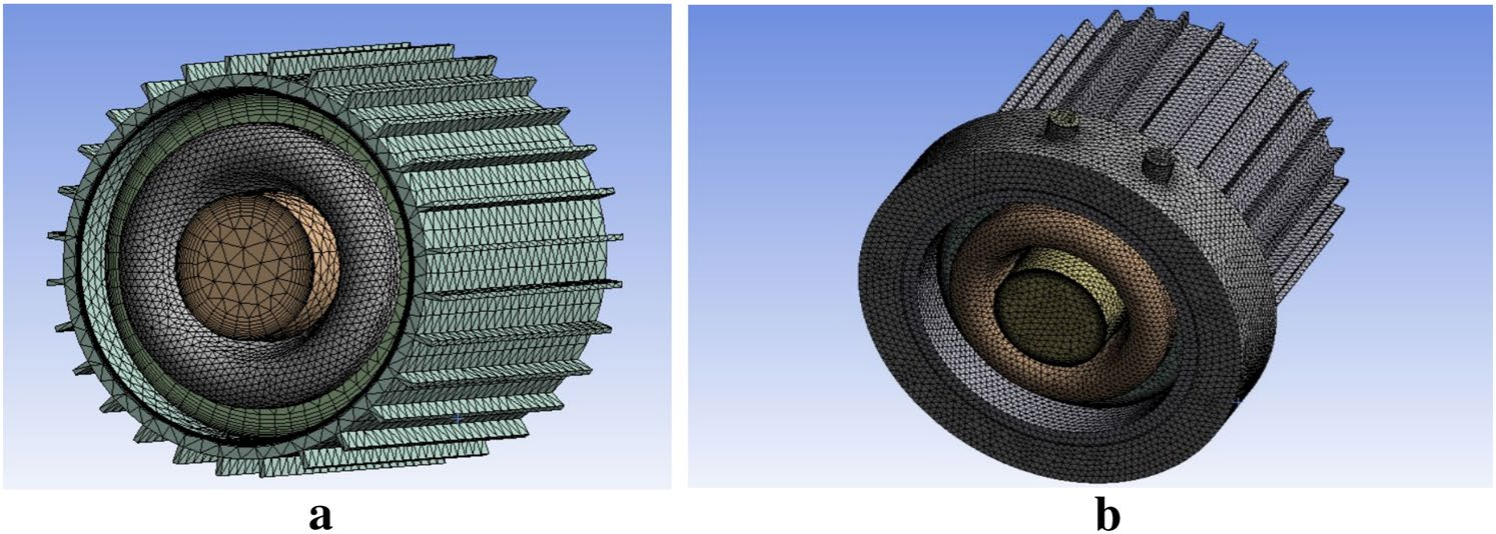
(**a**) Meshed structure of the air-cooled IM. (**b**) Meshed structure of the air- and water-cooled IM.

The IM model is simulated using CFD with FEM numerical technique. A mesh is built in CFD to partition the domain into a definite number of components for which solutions may be found. The tetrahedron mesh with appropriate element size is used in the overall complex geometry of motor components. 10-layered inflation is used at all interfaces to gather accurate results of heat transfer at this surface. The meshed geometry of both models of IM has been depicted in Fig. 6a,b.

#### Simulations

**Figure 7:**
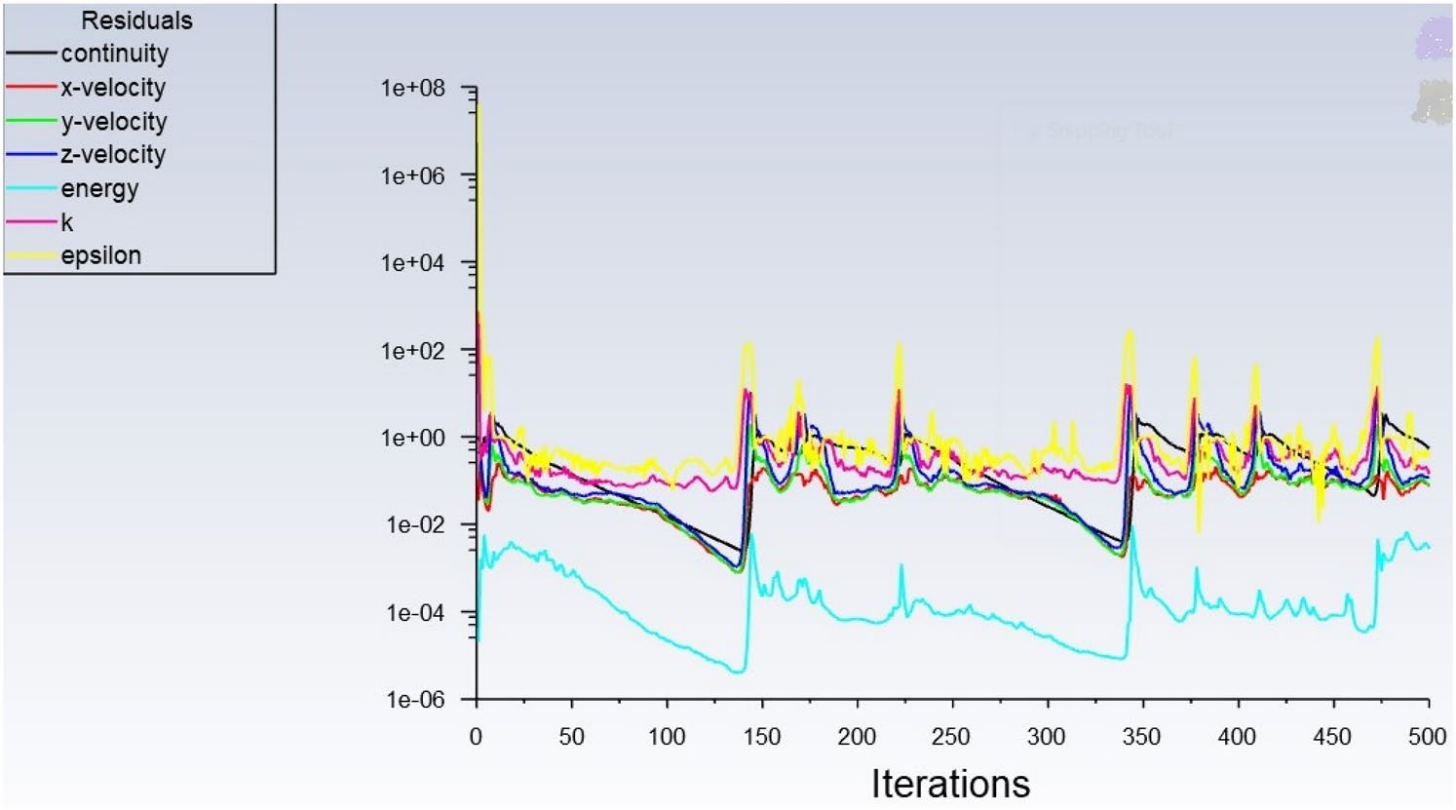
Numerical simulation results of the motor model performed for 500 iterations.

The energy equation enables to study of the heat transfer across different regions of the motor. The *K*-epsilon turbulent model with standard wall function is selected for modelling the turbulent flow around the outer surface. This model solves for kinetic energy (*E*_*k*_) and turbulent dissipation (epsilon). Copper, aluminium, steel, air and water are selected with their standard properties for utilization in respective areas. The heat generation rates (see Table 2) are given as input and different cell zone conditions are set^15,17,28,32^. The air velocity over the motor casing is set at 10 m/s for both the motor models and in addition, for the water jackets, three different water flow rates (5 LPM, 10 LPM and 15 LPM) are also considered. The residual for all equations is set to 1 × 10^–6^ for better accuracy. SIMPLE algorithm (Semi Implicit Method for Pressure Linked equation) is chosen to solve the Navier–Strokes (NS) equation. Upon completion of hybrid initialization, the setup is executed for 500 iterations as shown in Fig. 7.

**Table 2:** The heat generation rate of the IM.

Part	Heat generation rate (W)
Stator	2688
Rotor	2352
Winding	1680
Total	6720

## Results and discussion

A large capacity 100 kW air-cooled motor and an enhanced model of integrated air and water-cooled IM were modelled using SolidWorks 2017 software and further numerically simulated using ANSYS Fluent software. The heat generation rates were found through an extensive survey of existing scientific literature available on a 100-kW IM. Based on it, the heat generation rates were finalized and uniformly applied to both models of the motor. A uniform airflow velocity at 10 m/s was applied to both these models. Additionally for the integrated air- and water-cooled model, three different liquid flow rates were applied namely 5 LPM, 10 LPM and 15 LPM. Both these models were respectively examined and compared for their thermal performance based on temperature distribution. Additionally, for the integrated air and water-cooled model, the pumping power required to pump the water in the cooling jacket was also calculated.

### Thermal analysis of air-cooled IM

**Figure 8:**
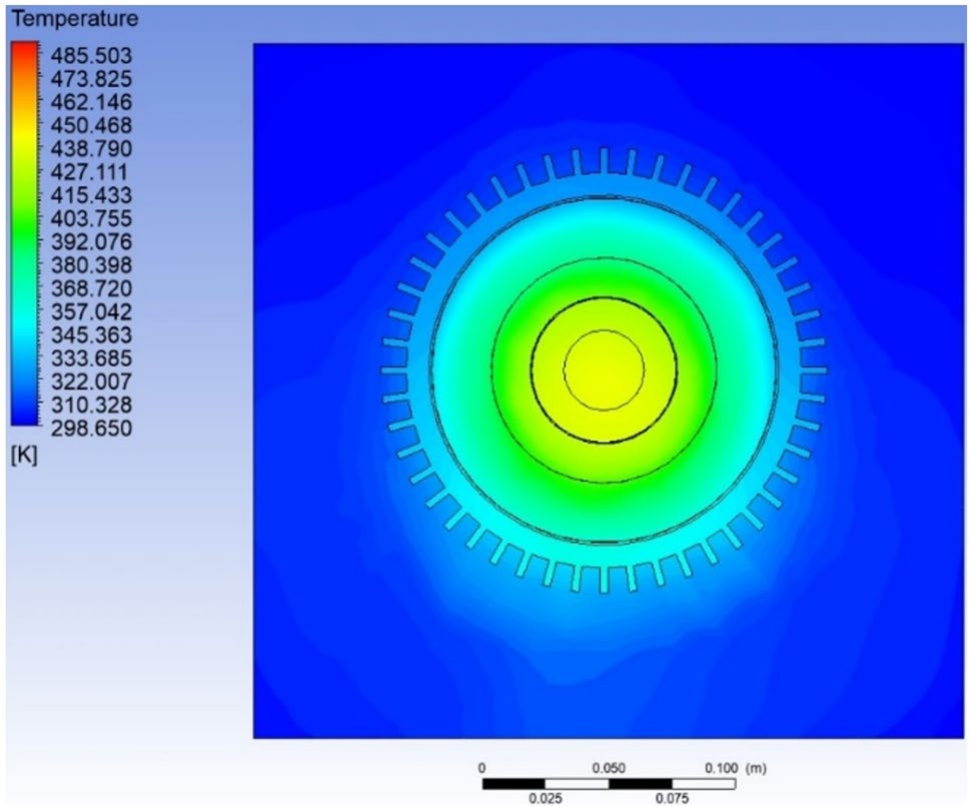
Temperature contour depicting variation across the cross-section of the motor.

**Figure 9:**
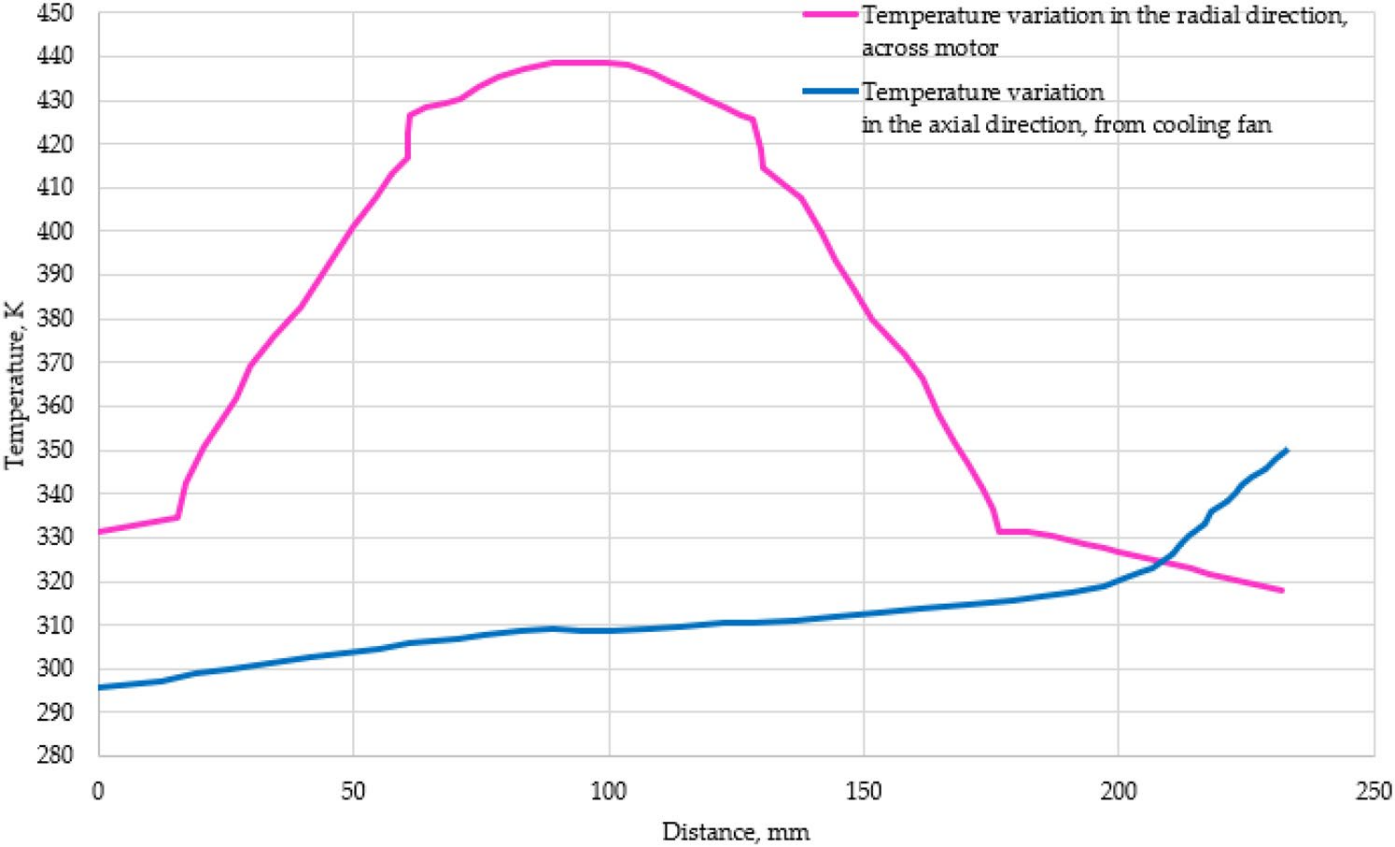
Temperature variation in an air-cooled motor.

The temperature variation along the cross-section of the IM was visualized with the help of the temperature contour (Fig. 8). It was observed that with the provided heat generation rate, the highest temperature occurs at the rotor surface. It could be noted that there is a uniform decrease in temperature when moving outwards in the radial direction, rotor–stator–outer surface (Fig. 9). This was a common observation in much of the literature surveyed on the similar capacity motor, wherein the highest temperature was noted in the rotor region. The highest temperature obtained in this study was in the rotor at 438 K (165 °C) while for the stator the highest temperature is observed to be 392 K (119 °C). The average temperature of the rotor is 435 K (162 °C) while for the stator, it was calculated to be 380 K (107 °C). The quantitative comparison has been depicted in Table 3.

**Table 3:** Temperature comparison between rotor and stator.

Component	Highest temperature (°C)	% Relative difference	Average temperature (°C)	% Relative difference
Rotor	165	38.65	162	51.4
Stator	119		107	

**Figure 10:**
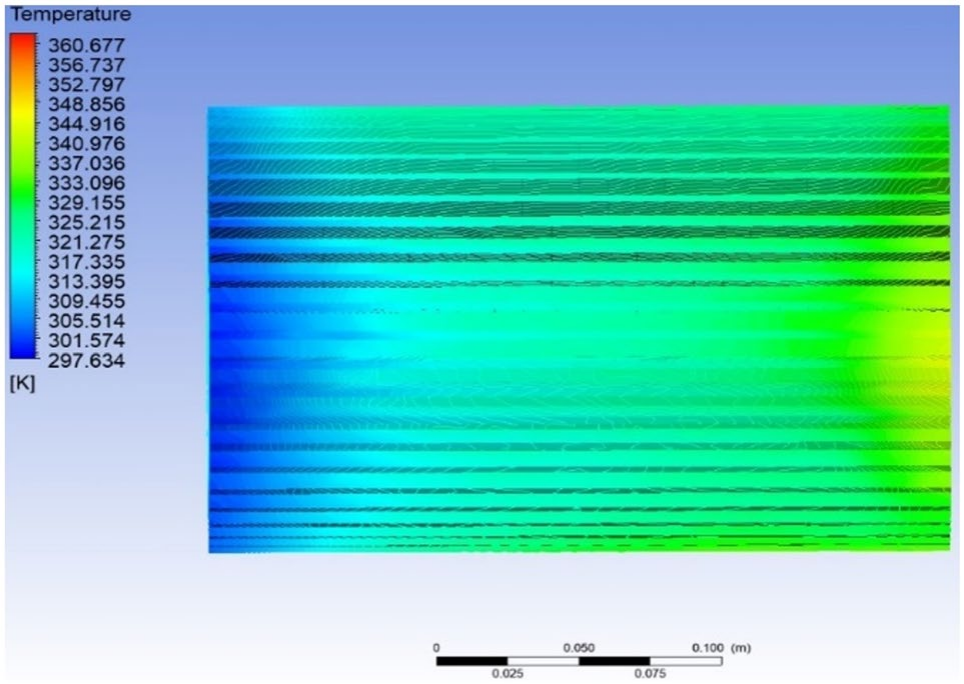
Temperature variation along the fin surface in the axial direction of the motor inside.

Results indicated that there is a subsequent rise in temperature along the surface of the motor casing on moving away from the location of the cooling fan (Fig. 10). This is expected since the air gets heated on moving along the surface of the motor. As the temperature of the air increases, its ability to transfer heat to the surroundings decreases due to the decrease in the temperature difference between the air and the motor casing. It is observed that the temperature of the fin near the air inlet is having an average temperature of 297 K (23.85 °C). The air upon passing further over the fin surface gets heated and the maximum temperature of 350 K (77 °C) is seen towards the right side of the motor casing which is away from the air entry point. This indicates a 17.84% temperature rise between both ends of the casing. Further, it is also observed that there is a jump, abrupt growth in the temperature from 320 to 350 K in the region where the stator winding is located (Fig. 9).

The rate of heat transfer depends on the difference in the temperature between the air and the outer fin surface. As the air travels over the fin surface, it gets heated up and as a result, the temperature difference decreases. This reduced heat transfer rate and heat released from the windings are responsible for the steep rise in the temperature towards the end surface.

The air velocity is reduced from the initial velocity of 10 m/s to around 1.8 m/s as it reaches the other side of the motor surface (Fig. 12). It is observed that the velocity of forced air reduces very fast within the first 10–12 mm and then gradually decreases as we move away from the fan. The velocity can be directly related to the heat transfer capabilities of air since the Reynolds number varies directly with the velocity. Since the decrease in Reynolds number causes the air to be less turbulent thereby diminishing its impact to transfer heat away from the motor surface thereby leading to a temperature increase. This result is further strengthened by Eq. (9), wherein the Nusselt’s number is inversely proportional to the Reynolds number.

**Figure 11:**
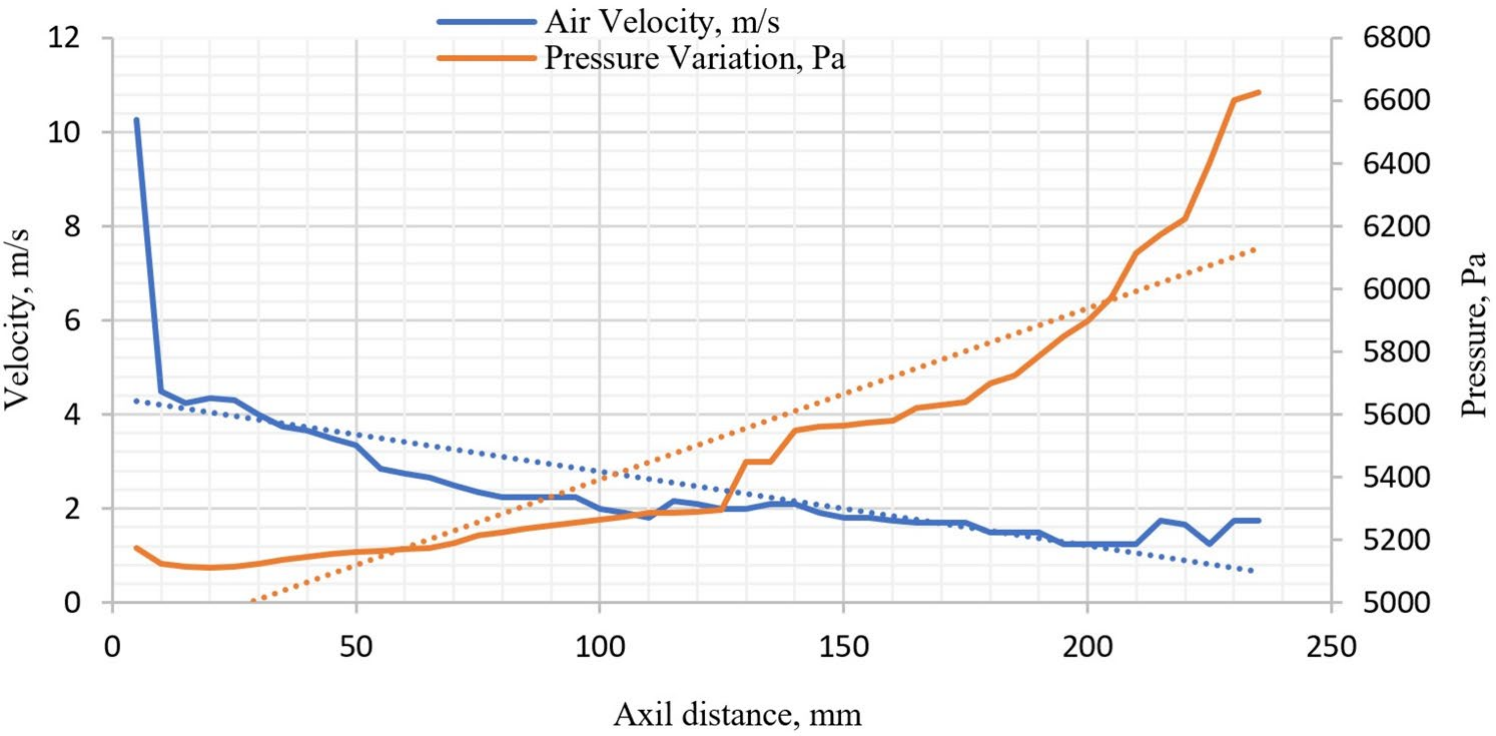
Velocity and pressure variation in the axial direction along the motor casing.

The pressure variation along the motor surface is depicted, see Fig. 11. The nature of the graph is similar to the temperature variation along the fin surface in the axial direction, see Fig. 9. The average values of variations in velocity and pressure in the axial direction along the motor casing are represented by dashed lines. The analysis indicate that the air pressure rises at the average rate 0.48 while the air velocity drops at the average rate of 0.24. This graph follows the Gay Lussac’s law wherein the temperature and pressure are directly proportional provided other parameters are kept constant. Since the pressure of the air depends upon the temperature of the fin surface and the convective heat transfer rate the pressure reduces steadily along the length of the IM. The pressure is minimum around the entry region of air and it increases similarly to the temperature to a maximum value towards the exit of the air over the motor surface, which is 28.37% more than the pressure at the entry region.

### Temperature validation for conventional air-cooled IM

The temperature of the stator and rotor is obtained using a 3-D numerical analysis through computational electromagnetic-thermal coupled air-cooled modeling on a 100-kW IM to analyse the performance. The investigations were performed to analyse the efficacy of the air gap fans in cooling the motor parts such as rotor, winding, stator, and frame^32^. These results were compared with the results obtained in this study and a % relative deviation was calculated to check the accuracy of the results obtained. It was observed that a maximum deviation of 10.73% was noted in the rotor temperature. Since the results obtained were around the 10% limit, it is hence thermally validated (Table 4).

**Table 4:** Temperature validation of obtained results.

	Average temperature (K) obtained by Chiwon et al.	The average temperature (K) was obtained in this study	% Relative deviation
Rotor	485	438	10.73
Stator	387	392	1.29

### Thermal analysis of integrated air- and water-cooled IM

The thermal analysis of the integrated air- and water-cooled IM were carried out using the same heat generation rates used for the conventional air-cooled IM. The integrated model in addition consists of a water jacket and a pump. The cooling liquid was fixed as water and three different water flow rates namely 5 LPM, 10 LPM and 15 LPM were considered. The flow path consisted of one inlet and one outlet having a single pass. The pumping power for all 3 flow rates was calculated using the pressure drop from the inlet to the outlet. The pressure drop was multiplied by the discharge (in m^3^/s) and was further divided by the pump efficiency which was assumed at 90%. An increase in the flow rate increases the heat transfer coefficient improving the heat transfer rate from the motor. The graphs obtained for all three flow rates were similar, increasing linearly on moving away from the air inlet side. However, the maximum temperatures obtained were different for the three different flow rates.

**Figure 12:**
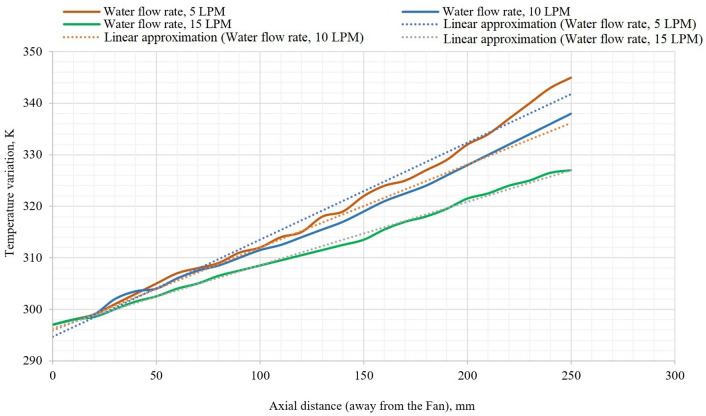
Temperature variation along fin for the coolant flow rates, 5 LPM, 10 LPM, and 15 LPM.

At a flow rate of 5 LPM temperature along the fin is linearly increasing, which was similar to that obtained for an air-cooled motor (Fig. 12). However, the maximum temperature observed was 340 K which was 10 K lower (350 K) than the air-cooled motor. This was a reduction of 2.94% in the maximum temperature which was achieved with an additional power requirement of 2.7 W used to drive the water around the water jacket.

For a flow rate of 10 LPM, the temperature along the fin is linearly increasing which was similar to that obtained for the air-cooled motor (Fig. 12). However, when compared to the flow rate of 5 LPM, the maximum temperature, in this case, was slightly lower (335 K) compared to 340 K for 5LPM. When compared to an air-cooled IM, the maximum temperature was reduced from 350 to 335 K which was a reduction of 4.47%. This came at the cost of an additional 5.2 W of pumping power.

On investigating different available scientific literature on heat transfer using forced convection, it was a common observation that as the flow rate of the fluid is increased, the heat transfer is also increased provided rest with all parameters are kept constant. A similar observation indicated that at 15 LPM, which is also the highest flow rate considered, the maximum temperature is the lowest among all the models considered (326 K) (Fig. 12). There was a reduction of 7.36% as compared to the air-cooled motor. The pumping power required to achieve this is also the highest which is 8.1 W. The dashed lines represent the average rate of change of temperatures along the fin of the casing, in axial direction, for various rates of water flow. Investigations revealed that the average rate of rise in temperature is 0.5 for the water flow rate of 5 LPM, 0.4 for the water flow rate of 10 LPM and it is 0.3 for the flow rate of 15 LPM. This clearly indicates the fall in rate of rise in temperature with the rise in water flow rate.

**Figure 13:**
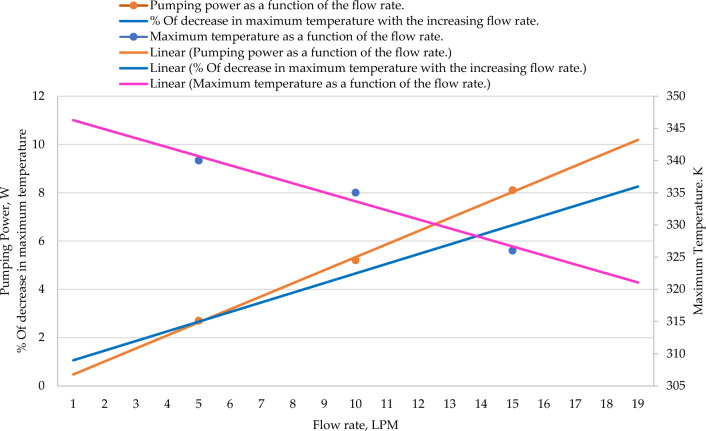
Pumping power, maximum temperature, and % Of decrease in maximum temperature as a function of the flow rate.

Investigations revealed that there was a substantial decrease in the maximum temperature that reached the motor surface. These results were as expected from the literature review since all the literature related to liquid-cooled systems pointed out a common conclusion that by increasing the flow rate, the heat transfer characteristics improved. The maximum temperature reduced from 340 K at a flow rate of 5 LPM to 326 K at a flow rate of 15 LPM (Fig. 13).

It is also a common observation that on increasing the flow rate, the pumping power will also increase which is pointed out clearly in Fig. 13. An almost linear graph is obtained since the flow rate is increased proportionately keeping all the other parameters constant.

**Figure 14:**
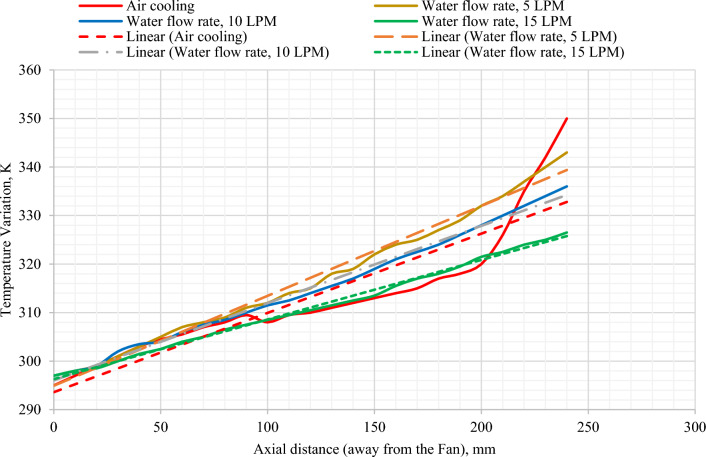
Temperature variation axially with air-cooled IM and integrated air- and water-cooled IM.

The average changes in the temperature axially are represented with the dashed lines. But the rate of drop in temperature with the water cooling is phenomenal compared to the air cooling, especially at the farthest point from the fan. Investigations reveal that in the first 100 mm distance from the fan, all the cooling schemes appear to be similar. For the next 100 mm distance air cooling seems to be better than the integrated approach (Fig. 14). The reason for this could be uneven temperature in the motor axially from the fan. Whereas, the ineffectiveness of air cooling is visible in the last section of the motor due to stator end winding temperature. This is not at all desirable with respect to the safety and longevity of the motor is concerned. But the advantage of the integrated method is there is uniform rate of rise in the in the temperature which is desirable for the safe operation of the motor.

The % of the decrease in the maximum temperature, when compared to the conventional air-cooled IM on increasing the flow rate. An upward partly linear trend was observed with a % decrease of 7.69% at a flow rate of 15 LPM. The trends of the effectiveness of cooling with an air-cooled IM and integrated air- and water-cooled IM validates the usefulness of this research (Fig. 13). Comparison between air-cooled IM and integrated air- and water-cooled IM clearly indicates the maximum temperature reduction for various coolant flow rates (Table 5).

**Table 5:** Comparison between air-cooled IM and integrated air- and water-cooled IM.

	Air-cooled IM	Integrated air- and water-cooled IM		
Coolant (water) flow rate	–	5 LPM	10 LPM	15 LPM
Max. temperature (K)	350	340	334	325
Air velocity (m/s)	10	10	10	10
% of reduction in maximum temperature (%)	–	2.94	4.79	7.69
Pumping power (W)	–	2.70	5.20	8.10

## Conclusions

The presented work has successfully numerically investigated the model of a 100-kW air-cooled IM and an improved thermal management model of the same IM wherein a combination of air-cooling and an integrated water-cooling systems have been used to achieve a significant improvement in the cooling intensity of the motor. ANSYS Fluent version 2021 was used to numerically investigate the integrated air and the water-cooled model, modelled in SolidWorks 2017, by providing the heat generation rates uses the finite element method. Results are compared with the air-cooled model with similar input parameters. The air-cooled model is validated with the external available published data in literature. The air velocity induced due to the cooling fan was set at 10 m/s. The pumping power requirement for the water flow rates 5 LPM, 10 LPM and 15 LPM was also calculated. The main obtained results in this work indicate that for a flow rate of 5 LPM, there was a 2.94% reduction in the maximum temperature of an integrated air- and water-cooled IM compared to the air-cooled IM. Similarly, there was a 4.79% drop in maximum temperature for a flow rate of 10 LPM and a 7.69% reduction for a flow rate of 15 LPM. Though the pumping power was seen increasing with the increase in the flow rate, the life span of the machine could be safeguarded with the drop in maximum temperature.

Future scope of this study will consist the traction induction motors with water cooling that have a high heating power density in the rotor. Therefore, a more effective rotor cooling system is required for such high-power motors. For example, in Tesla and Audi induction motors, the rotor is permanently liquid-cooled. In addition, there are problems with the heating of the motor bearing provides decreasing of their lifespan, which would be our next objective. Secondly, it would be more interesting for the traction machine designers to read an article about a new housing material for some real water-cooled high-power density traction motor with a cooling system close to systems used in practice. Perhaps this could be also done in our future work. Also, our future works would focus on the motor housing and cooling jacket focusing on reducing the thermal effects, contact resistance, new materials with improved thermal performance; and optimized channel geometries; and design for reducing the pressure drop losses, nanofluid-based cooling systems; and increasing the heat transfer on the convection surfaces.

## Data Availability

The datasets used and/or analysed during the current study available from the corresponding author on reasonable request.

## Abbreviations

CFD: Computational fluid dynamics
LPM: Liter per minute
IM: Induction motor
LPTN: Lumped parameter thermal network
LPCM: Lumped parameter circuit method
FEA: Finite element analysis
FEM: Finite element method
HTC: Heat transfer coefficient
RTD: Resistance temperature detectors
TEFC: Totally enclosed fan-cooled
PCM: Phase change material
